# Fatigue in Ankylosing Spondylitis Is Associated With Psychological Factors and Brain Gray Matter

**DOI:** 10.3389/fmed.2019.00271

**Published:** 2019-11-21

**Authors:** Ting Li, Ling Zhou, Hongbo Zhao, Jing Song, Xiuwen Wang, Shiyuan Liu, Huji Xu

**Affiliations:** ^1^Department of Rheumatology and Immunology, Shanghai Changzheng Hospital, The Second Military Medical University, Shanghai, China; ^2^Department of Radiology, Shanghai Changzheng Hospital, The Second Military Medical University, Shanghai, China; ^3^Beijing Tsinghua Chang Gung Hospital, School of Clinical Medicine, Tsinghua University, Beijing, China; ^4^Peking-Tsinghua Center for Life Sciences, Tsinghua University, Beijing, China

**Keywords:** ankylosing spondylitis, fatigue, neuropsychological factors, gray matter, MRI

## Abstract

**Background:** Ankylosing spondylitis (AS) is a rheumatic inflammatory disease with unknown etiology, and fatigue is one of the main systemic symptoms of AS. The aim of the current study was to explore the mechanism of AS-associated fatigue (ASF) from multiple aspects, including neuropsychological changes.

**Method:** A total of 120 AS patients and 78 age- and sex-matched healthy individuals were recruited into the study. Fatigue was assessed by the fatigue item of the Bath Ankylosing Spondylitis Disease Activity Index (BASDAI) and the Multidimensional Assessment of Fatigue (MAF) scale. Anxiety and depression were assessed by the Hospital Anxiety and Depression Scale (HADS). The cortical thickness and subcortical gray matter volume were assessed using a Philips Achieva 3.0 T TX MRI scanner.

**Result:** Of the 120 AS patients, 103 (85.8%) reported varying degrees of fatigue. Among these fatigue cases, 33 (32.0%) were in the severe fatigue group (BASDAI-Fatigue ≥ 5), and 70 patients (68.0%) were considered to be in the mild fatigue group (BASDAI-Fatigue > 0 but <5). The BASDAI, ASDAS-CRP, HAD-A, and HAD-D scores of AS patients in the severe fatigue group were all significantly higher than those of patients in the mild fatigue and non-fatigue groups (all, *P* < 0.05). The structural equation model suggested that AS activity triggered the occurrence of fatigue by inducing psychological change. Finally, head MRI imaging found that the left thalamus volume in AS patients with severe fatigue was significantly larger than that in non-fatigue AS patients and healthy controls (both, *P* < 0.05).

**Conclusion:** The study revealed neuropsychological factors involved in fatigue in AS.

## Introduction

Ankylosing spondylitis (AS) is a rheumatic inflammatory disease with unknown etiology. Pathologically, AS mainly involves the middle axial bone, as represented by sacroiliitis and enthesitis (1). Clinically, pain, morning stiffness, and fatigue are considered the most common symptoms in AS patients, of which fatigue is a main systemic symptom, with an incidence of 50–70% (2, 3). Fatigue is one important factor contributing to the unsatisfactory treatment outcome and poor quality of life, or even disability, in AS patients. At present, there is no effective treatment for AS-associated fatigue (ASF). Furthermore, it was reported that only 60% of pain and 35% of fatigue can be relieved in AS patients who received biological treatment. Therefore, clinically, how to improve the symptom of fatigue in AS patients remains a challenge.

The etiology of ASF is complex and remains elusive. Previous studies suggested that several factors, including physiology, psychology and behavior, may contribute to its pathogenesis (4). One study revealed that anti-depressants could improve symptoms in AS patients with chronic fatigue syndrome, indicating that psychological factors were associated with ASF (5). Other studies claimed inflammation and autoimmunity as the main causes to ASF (6, 7). Hence, the aim of the present study was to explore the potential mechanism of ASF by analyzing multiple types of data, including clinical indexes and head MRIs, from recruited AS patients.

## Methods

### Subjects

A total of 120 AS patients were recruited from the Department of Rheumatology and Immunology of Shanghai Changzheng Hospital (Shanghai, China) from July to December 2017. The AS diagnosis was made according to the 1984 modified New York Criteria (8). All patients with any other diseases that may cause fatigue, such as fibromyalgia, malignant tumors or other chronic diseases were excluded from this study. In addition, 78 age- and sex-matched healthy individuals were recruited as controls. Moreover, 10 AS patients with severe fatigue [Bath Ankylosing Spondylitis Disease Activity Index-Fatigue (BASDAI-Fatigue) ≥ 5], 10 AS patients without fatigue (BASDAI-Fatigue = 0), and 6 age- and sex-matched healthy individuals were selected for magnetic resonance imaging (MRI) scanning and analysis. All patients participating in the study were older than 18 years old and provided written informed consent. The research protocol was approved by Shanghai Changzheng Hospital Ethics Committee (approval no 2017SL046).

### Data Collection

The clinical data of all participants were collected and summarized in Supplement Table 1. Likewise, the clinicopathological parameters of AS patients, including extra-articular manifestations, C-reactive protein (CRP) levels, Bath Ankylosing Spondylitis Disease Activity Index (BASDAI), Bath Ankylosing Spondylitis Functional Index (BASFI) score, CRP-based Ankylosing Spondylitis Disease Activity Score (ASDAS-CRP), were also recorded in Supplement Table 2.

### Measures of Clinical Variables

To assess the fatigue status of AS patients objectively and effectively, BASDAI scoring was used, which is the most commonly used method and has been verified by many studies (3, 9, 10). Briefly, a BASDAI-Fatigue score ≥ 5 was defined as severe fatigue, a BASDAI-Fatigue score > 0 but <5 was defined as mild fatigue, and a BASDAI-Fatigue score = 0 was defined as non-fatigue. In addition, considering that BASDAI-fatigue scoring could only assess fatigue from a single dimension, the Multidimensional Assessment of Fatigue (MAF), which can evaluate fatigue in AS patients from multiple dimensions, including the degree, severity, distress, the impact on life, and timing, was also used in this study (11, 12). Fatigue in healthy controls was also assessed by the MAF. Moreover, Cronbach's α coefficient was analyzed to determine the internal consistency of the MAF in assessing fatigue in AS patients. A value > 0.6 indicates good reliability, a value > 0.8 indicates very good reliability, and values > 0.9 represent excellent reliability.

Anxiety and depression were assessed by the Hospital Anxiety and Depression Scale (HADS) (13), which consists of 14 items and two dimensions to indicate anxiety or depression. Briefly, a score of 0–7 indicates no anxiety or depression; a score of 8–10 indicates the possibility of anxiety or depression; and a score of 11–21 indicates a high possibility of anxiety or depression.

Neuropathic pain was assessed by a pain detection questionnaire (PDQ) (14), where a score <12 indicates the absence of neuropathic pain; 19 > PDQ ≥ 12 indicates the possibility of neuropathic pain; and a PDQ score ≥ 19 indicates a high possibility of neuropathic pain.

### MRI

Head MRI data were acquired using a Philips Achieva 3.0T TX MRI scanner equipped with an 8-channel coil. A whole-brain 3D high-resolution anatomic scan was performed with a T1-weighted 3D Turbo field echo sequence (repetition time 8.1 ms, echo time 3.7 ms, 160 sagittal slices, voxel size 1 × 1 mm, matrix 256 × 256 pixels, field of view 240 × 240 mm, and flip angle 8). Cortical thickness and sub-cortical gray matter volume were calculated by FreeSurfer software (15, 16).

### Statistical Analysis

Statistical analysis was performed by SPSS16.0, and the normally distributed data are presented as the mean ± standard deviation (x ± SD). Normally distributed continuous variables were verified by two-sample *t*-tests, and analysis of variance was used for multi-group differences, and unordered categorical variables were verified by chi-squared tests. The structural equation model was analyzed by Amos 23.0.0, and the relationship between latent variables was analyzed by a structural equation model (SEM).

## Results

### General Condition of the Patients

According to Supplement Table 1, among the 120 AS patients, 104 were male, and 16 were female. The patients' ages ranged from 18 to 66 years old, with a mean age of 36.4 ± 10.5 years. Overall, the MAF-degree, MAF-severity, and MAF-timing fatigue scores in AS patients in the previous week were significantly higher than those in healthy controls (all, *P* < 0.01).

### Fatigue-Related Factors

After the assessment of fatigue, of the 120 AS patients, 103 (85.8%) reported varying degrees of fatigue. Among these fatigue patients, 33 (32.0%) were in the severe fatigue group (BASDAI-Fatigue ≥ 5), and 70 patients (68.0%) were considered to be in the mild fatigue group (BASDAI-Fatigue>0 but <5). As illustrated in Supplement Table 2, the demographic, social and disease-related data of the three groups were then analyzed. The BASDAI, ASDAS-CRP, anxiety (HAD-A), and depression (HAD-D) scores in the severe fatigue group were significantly higher than those in the mild fatigue and non-fatigue groups (all, *P* < 0.01).

**Figure 1:**
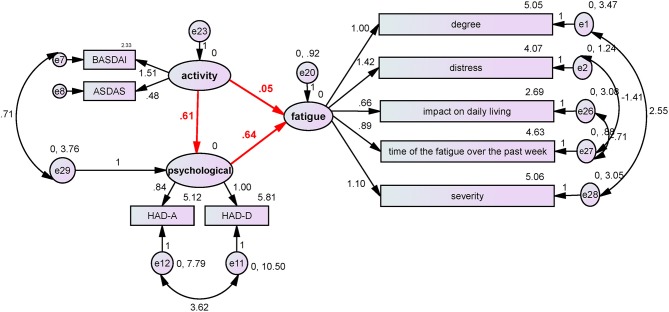
The influence of disease activity and neuropsychological factors on fatigue. BASDAI and ASDAS-CRP were used as the disease activity index, HAD-A and HAD-D were used as the neuropsychological index, and the five MAF items were used as the fatigue index. The structure equation model was successfully established (probability level = 0.211; CMIN/DF = 1.238; CFI = 0.992; NFI = 0.962; and RMSEA = 0.045). According to the SEM, disease hyperactivity could alter the psychological status (β = 0.61, *P* = 0.026), and the psychological status could induce the occurrence of fatigue (β = 0.64, *P* = 0.001), but disease activity did not directly induce the occurrence of fatigue (β = 0.05, *P* = 0.8). The results demonstrated that psychological factors played a critical role in the occurrence of ASF.

Using BASDAI and ASDAS-CRP as the disease activity index, HAD-A and HAD-D as the neuropsychological index, and the 5 MAF dimensions as the fatigue index, the SEMs of the indexes were finally analyzed. The model was successfully fitted (probability level = 0.211; CMIN/DF = 1.238; CFI = 0.992; NFI = 0.962; and RMSEA = 0.045). According to the SEM (Figure 1), disease hyperactivity could alter the psychological status (β = 0.61, *P* = 0.026), and the psychological status could induce the occurrence of fatigue (β = 0.64, *P* = 0.001), but disease activity did not directly induce the occurrence of fatigue (β = 0.05, *P* = 0.8). Collectively, these results demonstrated that psychological factors played a critical role in the occurrence of ASF.

Next, the Cronbach's α coefficient of the MAF was analyzed to determine the internal consistency of the MAF in assessing fatigue in AS patients. The results showed that the Cronbach's α coefficient of the MAF in AS was 0.90, suggesting that the MAF had a relatively high internal consistency and reliability.

### Imaging Findings

Ten severe AS patients with fatigue (F+), 10 non-fatigue AS (F–) patients and six healthy controls were chosen to participate in the MRI study. As shown in Supplement Table 3, there were no significant differences in age, sex, body mass index (BMI), disease duration, CRP, and PDQ between the groups (all, *P* > 0.05).

**Figure 2:**
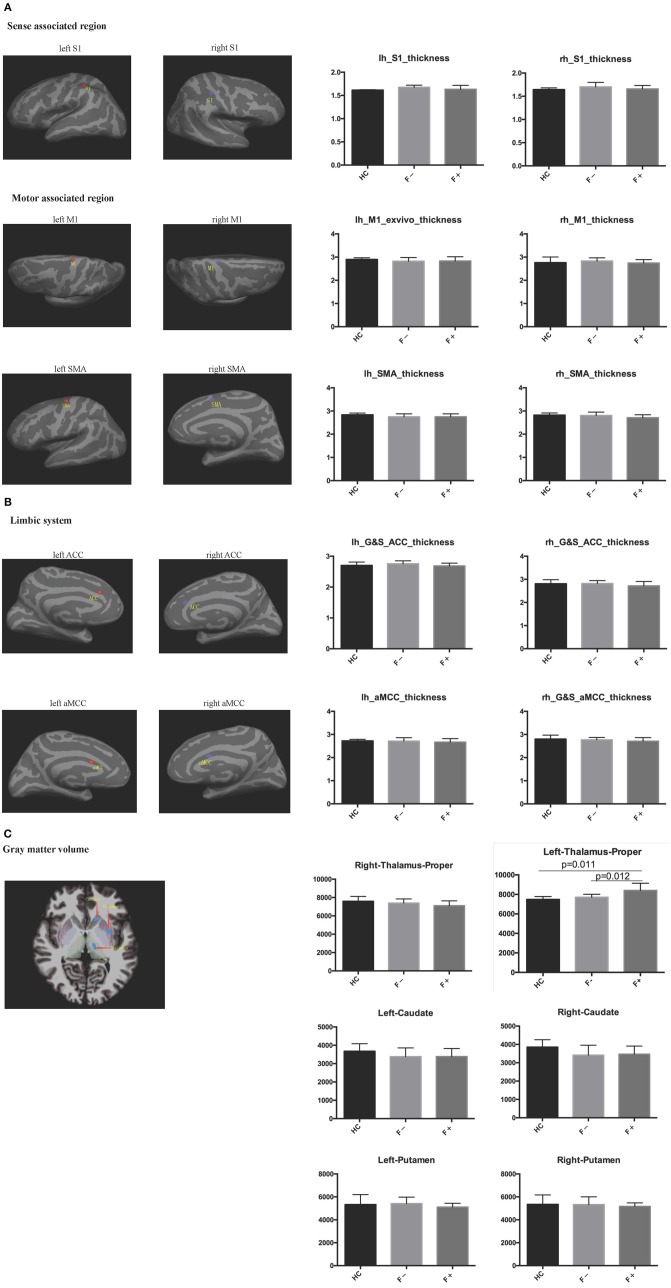
Cortical and subcortical gray matter segmentation was performed in head MRI. **(A)** Sense-associated regions and motor-associated regions; **(B)** Limbic system; **(C)** Gray matter volume. The left thalamus volume in AS patients with severe fatigue was significantly larger than that of non-fatigue AS patients and healthy controls.

To compare the cortical volume (including that of sense-associated regions, motor-associated regions and the limbic system) and subcortical gray matter volume (including that of the thalamus, putamen, and caudate) segmentation between the three groups, a head MRI scan was performed in these patients. The left thalamus volume of AS patients with severe fatigue (8,407 ± 739.4 mm^3^) was significantly larger than that in non-fatigue AS patients (7,708 ± 308.1 mm^3^) and healthy participants (7,481 ± 295.8 mm^3^) (both, *P* < 0.05; Figure 2). However, the differences in other indicators were not significant among the three groups.

## Discussion

Fatigue is a self-perception of persistent exhaustion and a decrease in physical and mental abilities, of which the characteristics include tiredness, lack of energy and motivation, and difficulty in concentrating attention on work or decision making (17). In AS patients, fatigue is one of the main systemic symptoms, occurring in 50–70% of cases (2, 3, 12, 18). In the present study, we found that 85.8% of AS patients exhibited varying degrees of fatigue symptoms, including 32.0% patients with severe fatigue. To date, the BASDAI-Fatigue score is the main indicator of ASF (19). However, this scoring system can only reflect a single dimension related to the degree of fatigue. Therefore, the MAF system, which can measure multiple other dimensions such as distress, impact on daily life activities, and timing of fatigue in the previous week, is also used to assess ASF (12) and, therefore, reflects the overall status of ASF in a more comprehensive way. In this study, the results revealed a correlation between the MAF dimensions of degree and severity and the BASDAI-Fatigue score. In addition, the Cronbach's α coefficient of the MAF was 0.90 in AS patients, suggesting that the MAF had a relatively high internal consistency and reliability.

Usually, the occurrence of fatigue in AS patients is considered to be associated with pathological factors, including the decreased ability of skeletomuscular activity due to inflammation, disease activity, and osteoporosis. However, some studies have discovered that complete relief of fatigue was achieved in only 35% of AS patients who received biomedical drugs (20). In our study, there was no significant difference in CRP levels between the severe fatigue, mild fatigue and non-fatigue groups, suggesting that there might be extra-inflammatory factors contributing to the occurrence of ASF, including psychological, social and demographic factors (21). Further study of the structural equation model showed that psychological changes played a key role in the occurrence of ASF. As such, we speculated that psychological changes might directly trigger the occurrence of fatigue. Alternatively, the impact of disease activity on ASF was through psychological changes. To some extent, this view was partly in line with the finding of Wu et al. (20) that residual fatigue was prominent after treatment with tumor necrosis factor inhibitors, suggesting that part of fatigue pathophysiology is unrelated to disease activity. Fatigue is the subjective perception of patients, which can be affected by pain, pressure, limited spinal mobility, and other factors in the assessment of fatigue in AS patients. Other studies have demonstrated that anti-depressants, including tricyclics and selective 5-HT reuptake inhibitors, could markedly improve the symptoms of patients with chronic fatigue syndrome (5, 22). Therefore, the exploration of whether mood regulatory drugs can be used to relieve the symptoms of ASF is warranted.

Fatigue can be classified as either peripheral or central, with peripheral fatigue mainly being related to decreased muscular tone or metabolic abnormalities and central fatigue mainly being related to structural or functional abnormalities of the spinal cord, cerebral cortex or subcortical gray matter; usually, these two forms of fatigue may co-exist in chromic disease-associated fatigue such as ASF (21). Using MRI, some researchers discovered that the cerebral gray matter, specifically areas related to attention and the somatosensory cortex, were thinner in AS patients with severe fatigue; meanwhile, the integrity of the cerebral white matter connecting the abovementioned related areas was reduced (23). Moreover, compared with healthy controls, the cortex in the primary somatosensory area, insular lobe, anterior cingulate and anterior cingulate cortex was thinner, while the volume of the gray matter of the thalamus and putamen was increased (24). In accordance with the previous studies of Wu et al. (23) and Andreasen et al. (25), our results in this study showed that the left thalamus volume in AS patients with severe fatigue was significantly larger than that in AS patients without fatigue and healthy controls. No significant changes were observed in the right thalamus volume or the thickness of the cerebral cortex, which was inconsistent with the findings in the study of Wu et al. (23). One possible reason for this inconsistency is the small sample size in our study. In addition, it has been reported that significantly higher activity in the left thalamus (26) and higher blood flow blood flow in the left thalamus (27) were observed in patients with chronic fatigue syndrome. Therefore, we suggest that the increased left thalamus volume in AS patients is of great significance. In addition, we did not find any significant change in the thickness of the cortex in the primary somatosensory area in AS patients, and we speculated that there was no significant difference in the pain detection score between AS patients with severe fatigue and those without fatigue. Previous studies also demonstrated that inflammation, chronic pain and enterobacteria could induce structural changes in the nervous system and neurotransmitters (28–33). However, few studies have reported structural and functional changes in the nervous system in AS, and the conclusions that they drew are not all consistent. In the present study, we demonstrated that clinical changes in the nervous system played an important role in the subjective symptoms of fatigue in AS patients. However, larger-sample studies or magnetic resonance spectroscopy (MRS) are necessary to verify structural and functional changes in the central nervous system of AS patients.

In conclusion, we analyzed multiple types of data, including clinical indexes and head MRI scans, from recruited AS patients and revealed neuropsychological factors contributing to fatigue in AS, which sheds new light on the pathogenesis of AS fatigue and could provide novel therapeutic strategies for ASF. However, despite these findings, there are still some limitations to our study. For instance, decreased muscle strength and limited spinal joint mobility are very common symptoms in AS patients that may affect the assessment of fatigue in these patients. However, methods for eliminating those effects are currently lacking. In addition, considering economic cost, operation difficulty and the large amount of data, a small number of AS patients were included in the head MRI study. Further studies with more samples should be carried out to verify and expand the research results in the future.

## Conclusion

In the current study, we found that the BASDAI, ASDAS-CRP, anxiety (HAD-A), and depression (HAD-D) scores of the severe fatigue group were significantly higher than those of the mild fatigue and non-fatigue groups. In MRI, we further found that the left thalamus volume in severe fatigue AS patients was significantly larger than that in non-fatigue AS patients and healthy participants. Taken together, our study suggests that neuropsychological factors play an important role in the pathogenesis of fatigue in AS.

## Data Availability Statement

All datasets generated for this study are included in the article/Supplementary Material.

## Ethics Statement

The studies involving human participants were reviewed and approved by Ethics committee of Changzheng Hospital. The patients/participants provided their written informed consent to participate in this study.

## Author Contributions

All authors were involved in drafting the manuscript, revising it critically for important intellectual content, and in the final approval of the publication.

### Conflict of Interest

The authors declare that the research was conducted in the absence of any commercial or financial relationships that could be construed as a potential conflict of interest.

## Abbreviations

AS: ankylosing spondylitis
ASF: ankylosing spondylitis-associated fatigue
MRI: magnetic resonance imaging
CRP: C-reactive protein
BASDAI: bath ankylosing spondylitis disease activity index
BASFI: bath ankylosing spondylitis functional index
ASDAS-CRP: CRP-based ankylosing spondylitis disease activity score
MAF: multidimensional assessment of fatigue
HADS: hospital anxiety and depression scale
PDQ (neuropathic pain assessment): pain detection questionnaire
SEM: structural equation model.
