# Supplementary material for: Fatigue in Ankylosing Spondylitis Is Associated With Psychological Factors and Brain Gray Matter
Source: Front Med (Lausanne). 2019 Nov 21;6:271. doi: 10.3389/fmed.2019.00271 (PMC6882166; doi:10.3389/fmed.2019.00271)
Supplement: Supplementary file 1 [file Data_Sheet_1.docx]

**Supplement table 1 General data of AS patients**

| Item | | AS  n=120 | HC  n=78 |
| --- | --- | --- | --- |
| Age (yr) | | 36.4±10.5 | 40.5±12.2 |
| Sex (male) | | 104（86.7%） | 62（79.5%） |
| Income | <50000 | 54（45%） | 27（34.6%） |
|  | 50000~100000 | 39（32.5%） | 28（35.9%） |
|  | 100000~200000 | 20（16.7%） | 16（20.6%） |
|  | >200000 | 7（5.8%） | 7（8.9%） |
| Education level | Primary school | 4（3.3%） | 0 |
|  | Junior school | 26（21.7%） | 8（10.3%） |
|  | High school | 33（27.5%） | 12（15.4%） |
|  | College | 49（40.8%） | 48（61.5%） |
|  | Master or higher degree | 8（6.7%） | 10（12.8%） |
| Disease duration (yr) | | 10.6±8.5 |  |
| Smoking | | 40（33.3%） | 22 (28.2%) |
| Extra-articular manifestations | Uveitis | 17（14.2%） |  |
|  | IBD | 3（2.5%） |  |
|  | Psoriasis | 4（3.3%） |  |
|  | No | 96（80%） |  |
| CRP (mg/L) | | 13.6±16.8 |  |
| HLA-B27 (positive) | | 100% |  |
| BASDAI | | 2.3±1.9 |  |
| ASDAS-CRP | | 1.5±0.8 |  |
| MAF-degree | | 5.14±2.57** | 2.75±2.24 |
| MAF-severity | | 5.13±2.58** | 2.96±1.98 |
| MAF-distress | | 4.07±2.76 | 3.21±2.25 |
| MAF-impact on activities of daily living | | 2.48±1.93 | 1.95±0.86 |
| MAF-timing of the fatigue over the past week | | 4.67±1.82** | 2.86±1.77 |

IBD, inflammatory bowel disease; MAF, multidimensional assessment of fatigue; BASDAI, Bath Ankylosing Spondylitis Disease Activity Index; ASDAS-CRP, Ankylosing Spondylitis Disease Activity Score-CRP. **P＜0.01

**Supplement table 2 Analysis of related factors in severe fatigue, mild fatigue and non fatigue patients**

|  | | Severe Fatigue (*n*=33) | Mild Fatigue  (*n*=70) | Non fatigue  (n=17) |
| --- | --- | --- | --- | --- |
| Age (yr) | | 36.7 ± 10.9 | 35.7 ± 9.6 | 38.0±13.4 |
| Sex | M | 28 | 63 | 15 |
|  | F | 5 | 7 | 2 |
| Disease duration (yr) | | 10.71±9.0 | 10.0±7.2 | 12.9±12,3 |
| Income | <50000 | 20 | 30 | 5 |
|  | 50000~ | 9 | 22 | 9 |
|  | 100000~ | 3 | 12 | 3 |
|  | >200000 | 1 | 6 | 0 |
| Education level | Primary school | 1 | 3 | 0 |
|  | Junior school | 7 | 16 | 3 |
|  | High school | 11 | 15 | 7 |
|  | College | 12 | 30 | 7 |
|  | Master or higher degree | 2 | 6 | 0 |
| Smoking (cigarettes/yr) | | 68.1 ±119.6 | 80.2 ± 209.7 | 50.6±75.0 |
| CRP | | 12.1±10.9 | 12.7±15.3 | 19.8±28.7 |
| BASDAI | | 4.5 ± 1.8** | 1.8 ±1.3 | 0.6±1.1 |
| ASDAS-CRP | | 2.2 ± 0.9** | 1.3± 0.7 | 0.9±0.4 |
| Sleeping length (hr) | | 6.63 ± 1.43 | 7.04 ± 1.16 | 7.53±1.46 |
| Anxiety (HAD-A) | | 6.5 ± 3.0** | 4.9± 3.5 | 3.1±2.6 |
| Depression (HAD-D) | | 7.8 ± 4.0** | 5.4 ± 3.6 | 3.9±3.6 |
| Working hours | | 33.8 ± 26.8 | 35.9± 24.8 | 31.1±21.2 |
| MAF-degree | | 6.87±1.81** | 4.60±2.49 | 3.58±2,42 |
| MAF-severity | | 6.60±1.98** | 4.68±2.64 | 3.76±2.16 |
| MAF-distress | | 5.63±2.95** | 3.67±2.48 | 2.82±2.09 |
| MAF-impact on daily activities | | 3.26±1.67** | 2.37±2.12 | 1.82±1.54 |
| MAF-timing of fatigue over the past week | | 5.85±1.35** | 4.38±1.82 | 3.35±1.27 |

BASDAI, Bath Ankylosing Spondylitis Disease Activity Index; ASDAS-CRP, Ankylosing Spondylitis Disease Activity Score-CRP; HAD, Hospital Anxiety and Depression Scale; MAF, multidimensional assessment of fatigue. **P<0.01

**Supplement table 3 General data of ASF+ patients, ASF- patients and healthy controls**

|  | F+  (*n*=10) | F-  (*n*=10) | HC  (*n*=6) |
| --- | --- | --- | --- |
| Age | 42.1±10.1 | 38.4±10.4 | 35.6±5.7 |
| Sex | All males | | |
| BMI | 24.6±4.3 | 21.7±2.6 | 23.7±3.6 |
| Disease duration | 11.5±9.9 | 13.3±10.7 |  |
| CRP | 11.4±6.7 | 11.9±13.4 |  |
| Pain detect | 11.7±6.3 | 10.3±6.9 |  |

*P<0.05, **P<0.01
